# A DREB-Like Transcription Factor From Maize (*Zea mays*), ZmDREB4.1, Plays a Negative Role in Plant Growth and Development

**DOI:** 10.3389/fpls.2018.00395

**Published:** 2018-04-04

**Authors:** Shixue Li, Qian Zhao, Dengyun Zhu, Jingjuan Yu

**Affiliations:** State Key Laboratory of Agrobiotechnology, College of Biological Sciences, China Agricultural University, Beijing, China

**Keywords:** DREB, *ZmDREB4.1*, transcriptional activator, dwarfism, maize, tobacco

## Abstract

The DREB (dehydration-responsive element binding)-type transcription factors are classified into six subgroups, named A-1 to A-6. The members of DREB A-1 and A-2 subgroups have been reported to be involved in response to various abiotic stresses. However, there were only a few genes belonging to A-3 to A-6 subgroups to be reported. In this study, we cloned a DREB A-4 subgroup gene from maize (*Zea mays*), *ZmDREB4.1*, and analyzed its characteristics and functions. *ZmDREB4.1* was expressed in roots, stems, and leaves at very low levels. It was not induced by any biotic or abiotic treatment. ZmDREB4.1 was located in the nucleus, could directly bind to the DRE element and functioned as a transcriptional activator. The constitutive expression of *ZmDREB4.1* in tobacco (*Nicotiana tabacum* L.) repressed leaf extension and hypocotyl, petiole and stem elongation. In maize, overexpression of *ZmDREB4.1* repressed calli growth and regeneration. Further analysis showed that the smaller leaves of transgenic tobacco resulted from inhibition of cell division. The contents of cytokinin and auxin in transgenic leaves were severely decreased. The shorter hypocotyls, stems and petioles of transgenic tobacco were caused by inhibition of cell elongation. The transgenic hypocotyls, stems and petioles contained reduced gibberellin levels. Application of exogenous GA3 rescued the shorter hypocotyls, stems and petioles, but not the smaller leaves. These results demonstrated that *ZmDREB4.1* plays an important role in the negative regulation of plant growth and development.

## Introduction

The APETALA2 (AP2)/ethylene-responsive factor (ERF) family is unique to plants. The transcription factors of AP2/ERF family possess one or two AP2 DNA-binding domains comprised of approximately 58 residues (Nakano et al., 2006; Velasco et al., 2010). The first cDNA encoding AP2/ERF protein, *APETALA2*, was identified from Arabidopsis (Jofuku et al., 1994). Since then, 147, 164 and 167 AP2/ERF genes have been identified from Arabidopsis, rice and maize, respectively. These genes are classified into four subfamilies, DREB, ERF, AP2, and RAV, according to the number of copies of the AP2 domain and their sequence similarity (Zhuang et al., 2010). Extensive studies have shown that AP2/ERF genes play important roles in plant flower (Elliott et al., 1996), seed (Ohto et al., 2009), and root (Kitomi et al., 2011) development, in response to biotic (Zhang et al., 2008) and abiotic (Stockinger et al., 1997) stresses and in the regulation of cell division (Mehrnia et al., 2013).

The DRE element was first identified in the *rd29A* promoter, which contains a DRE core sequence (ACCGAC) (Yamaguchi-Shinozaki and Shinozaki, 1994). The DREB-type transcription factors DREB1A and DREB2A were first identified using yeast one-hybrid screening by the DRE element (Liu et al., 1998). The DREB transcription factors were classified into six subgroups, named A-1 to A-6, based on sequence similarities of their AP2 domains (Sakuma et al., 2002). Previous studies have shown that most DREB1-type and DREB2-type genes are involved in abiotic stress responses (reviewed by Mizoi et al., 2012). Most *DREB1/CBF* genes are involved in response to low temperatures in plants (Akhtar et al., 2012). Overexpression of *DREB1B/CBF1* or *DREB1A/CBF3* enhanced cold and dehydration tolerance in transgenic Arabidopsis (Stockinger et al., 1997; Gilmour et al., 2000). DREB2-type genes were shown to be induced by osmotic stresses, such as drought and high salinity. Overexpression of an active form of *DREB2A* (*DREB2A-CA*), whose negative regulatory region was deleted, improved transgenic Arabidopsis tolerance to drought (Sakuma et al., 2006). In addition, several DREB A-1 or A-2 subgroup genes, *SiARDP* from foxtail millet (Li et al., 2014), *ZmDREB2A* from maize (Qin et al., 2007), *OsDREB1A* and *OsDREB2B* from rice (Dubouzet et al., 2003), *GmDREB2* from soybean (Chen et al., 2007) and *PeDREB2* from populous (Chen et al., 2009) were also reported.

Several genes belonging to A-3 to A-6 subgroups were also reported to function in response to abiotic stress or plant growth and development. Overexpression of *GhDBP3* (Huang and Liu, 2006), *MnDREB4* (Liu et al., 2015) and *StDREB1* (Bouaziz et al., 2013) of DREB A-4 subgroup; *PeDREB2a* (Zhou et al., 2012), *StDREB2* (Bouaziz et al., 2012) and *ScDREB8* (Liang et al., 2017) of DREB A-5 subgroup; and *GhDBP2* (Huang et al., 2008) and *SsDREB* (Zhang et al., 2015) of DREB A-6 subgroup can improve plant tolerance to abiotic stresses. *ABI4*, a member of DREB A-3 subgroup, was identified from Arabidopsis. It is involved in abscisic acid (ABA) signal transduction and seed development (Finkelstein et al., 1998; Söderman et al., 2000). The *ABI4* homologous gene from maize was also identified and analyzed (Niu et al., 2002). *TINY* identified from Arabidopsis was classified into the DREB A-4 subgroup. Its expression was induced by both abiotic stresses (drought and cold) and biotic stresses (ethylene and methyl jasmonate). *TINY*-overexpressing Arabidopsis displayed a partial constitutive ethylene triple response and dwarf phenotype (Wilson et al., 1996).

Plant morphology is affected by internal signals and external environmental stimuli. Plant hormones are a large class of small signal molecules and play an important role in the regulation of multiple aspects of plant growth and development (Seif El-Yazal et al., 2015). Gibberellic acids (GAs) have been reported to be involved in the regulation of cell elongation and division, stem elongation and seed germination (Richards et al., 2001). *GA20ox*, *GA3ox* and *GA2ox* are three major oxidase gene families that participate in GA synthesis and degradation (Hedden and Phillips, 2000). The levels of endogenous GAs are homeostatically modulated by negative feedback regulation of the *GA20ox* and *GA3ox* genes and positive feedforward regulation of *GA2ox* genes (Chiang et al., 1995; Xu et al., 1995; Cowling et al., 1998; Thomas et al., 1999; Hedden and Phillips, 2000). Many mutants that are deficient in GA biosynthesis or GA response exhibit a dwarf phenotype, such as *ga4*, *ga5* (Talon et al., 1999) and *gid1a gid1b gid1c* mutants (Griffiths et al., 2006). Cytokinins (CKs) were first identified for their ability to promote cell division in cultured plant cells (Miller et al., 1955). They are involved in regulation of the apical meristem maintenance, seed germination, flower and fruit development and leaf senescence (Mok and Mok, 2001). Many genetic and molecular biological studies have revealed the two-component regulatory system of CK perception and signal transduction (Hwang et al., 2002). In addition, it is reported that some components of CK signaling are involved in abiotic stress response (Tran et al., 2007).

In the present study, *ZmDREB4.1* belonging to the DREB A-4 subgroup was cloned from maize. *ZmDREB4.1* is a constitutively expressed gene, even though its expression level is very low. It is not induced either by abiotic (drought, salt, or cold) or by biotic (wound, NAA, 6-BA, GA, or ACC) stresses. ZmDREB4.1 has the characteristics of a typical transcription factor. It is located in the nucleus of maize protoplasts and young stem tissue, specifically bound to the DRE element, and activates the expression of downstream genes. We analyzed its function by overexpressing *ZmDREB4.1* in both tobacco and maize. *ZmDREB4.1*-overexpressing tobacco exhibited smaller leaves and shorter hypocotyls, petioles and stems. *ZmDREB4.1*-overexpressing maize calli grew slowly and failed to regenerate seedlings. Our work provides evidence that *ZmDREB4.1* plays a negative role in regulation of plant growth and development. In addition, its mechanisms were analyzed and discussed.

## Materials and Methods

### Plant Materials and Stress Treatments

Maize B73 inbred seeds were germinated on moist gauze overnight and the germinated seeds were then grown in mixtures of half vermiculite and half soil at 28°C under a long-day photoperiod (16 h light, 8 h dark) in an artificial illumination incubator with 70% relative humidity. The three-leaf seedlings were selected and cleaned. The seedlings were then fastened to plastic foam, transferred to 1/4 Hoagland solution and grown hydroponically with oxygen for 3 days. The 1/4 Hoagland solution was replaced every day during culture. The seedlings were then exposed to various treatments. For salt, drought, ABA, auxin, CK, GA and ethylene treatments, the seedlings were immersed in 1/4 Hoagland solution containing 100 mM NaCl, 20% PEG-6000, 10 μM ABA, 1 μM IAA, 1 μM 6-BA, 10 μM GA3, and 10 μM ACC, respectively. For cold treatment, seedlings in the soil were placed at 4°C. For wound treatment, the leaves were cut with a blade. The seedlings were collected after treatment and frozen in liquid nitrogen for RNA extraction. Meanwhile, seedlings without any treatment were used as controls.

For protoplast isolation, the germinated maize seeds were grown in vermiculite at 28°C under a long-day photoperiod (16 h light, 8 h dark) in an artificial illumination incubator with 70% relative humidity for 3 days and were then shifted to a completely dark artificial illumination incubator for 7–10 days until the second leaf was approximately 10–15 cm. The middle section (6–8 cm) of the second leaf was used for protoplast isolation.

The wild-type and *ZmDREB4.1*-overexpression tobacco seeds were sterilized by chlorine for 2 h and then grown on MS medium at 28°C under a long-day photoperiod (16 h light, 8 h dark) in an artificial illumination incubator with 70% relative humidity. The seedlings were used for RNA extraction and phenotype observation. For drought treatment, seeds were grown in mixtures of half vermiculite and half soil at 28°C under a long-day photoperiod (16 h light, 8 h dark) in a greenhouse and the germinated seedlings were not watered for 2 weeks, then rewatered and grew for 1 week.

### RNA Extraction and Quantitative PCR Analysis

Total RNA was extracted using TRIzol reagent (Ambion, United States), and first-strand cDNA synthesis was performed using 5 μg of total RNA and 20-bp poly (dT) oligonucleotide according to the protocol of the Reverse Transcription System (Promega, United States). Quantitative RT-PCR analysis was performed using the SYBR green method with the CWBIO Fast SYBR Mixture real-time PCR system (CWBIO, China). The primers used in this study were showed in **Table 1**. The expression levels of relevant genes were determined by the ΔΔ*C*T method.

**Table 1:** Primer sequences used in this study.

Primer name	Sequence (5′–3′)	Restriction sites
ZmDREB4.1-F	ATGGAGCACGAGCAGGTGTCG	
ZmDREB4.1-R	TCAATGTTCCCAAAGCAGCAAGG	
ZmDREB4.1-ORF-F	TCTAGAATGGAGCACGAGCAGGTGTCG	*Xba* I
ZmDREB4.1-ORF-R	GAGCTCTCAATGTTCCCAAAGCAGCAAGG	*Sac* I
ZmDREB4.1-qRT-F	CATTGGAGTTGGACTGGGCTATACG	
ZmDREB4.1-qRT-R	ATGGTCAATGTTCCCAAAGCAGCAA	
ZmDREB4.1-SL-F	TCTAGAATGGAGCACGAGCAGGTGTCG	*Xba* I
ZmDREB4.1-SL-R	CCCGGGATGTTCCCAAAGCAGCAAGGGCT	*Sma* I
ZmDREB4.1-TA-F	GAATTCATGGAGCACGAGCAGGTGTCG	*Eco*R I
ZmDREB4.1-TA-R	GTCGACTCAATGTTCCCAAAGCAGCAAGGG	*Sal* I
ZmDREB4.1-YH-F	GAATTCATGGAGCACGAGCAGGTGTCG	*Eco*R I
ZmDREB4.1-YH-R	GGATCCTCAATGTTCCCAAAGCAGCAAGG	*Bam*H I
ZmDREB4.1-PE-F	GAATTCCTCAACGGCTCGTCGTCGCC	*Eco*R I
ZmDREB4.1-PE-R	GTCGACTCAAGCCGGCACAGTGGCCGC	*Sal* I
mini35S-F	AAGCTTATCTCCACTGACGTAAGGGATG	*Hin*d III
mini35S-R	CCATGGCGTGTTCTCTCCAAATGAAATG	*Nco* I
DRE element	GATATACTACCGACATGAGTTC	
mDRE element	GATATACTAAAAAAATGAGTTC	
IPT3-qRT-F	CCAAGAACTTCTGTGACATGGCTTCA	
IPT3-qRT-R	ACCTCGTTGACAAGTCCTTTCTCGA	
IPT5-qRT-F	AAGCAGAGGTTGTGAACAGTGACAA	
IPT5-qRT-R	TCGACCCTCCAGCAATTATCGGTAG	
YUCCA9-qRT-F	TTATTGTAGGTGCAGGTCCATCGGG	
YUCCA9-qRT-R	TGTCTCTTTGTTGGGTACTCAGGGT	
YUCCA10-qRT-F	TTCGATGCCCAAAACATAGCCACAG	
YUCCA10-qRT-R	AATCACATCCGATCGACCCCTTCAT	
YUCCA11-qRT-F	AGGATTAGATGGCTTTGGTGGGACA	
YUCCA11-qRT-R	ACAGGGCTACGAACAATAATGGAGGT	
GA2ox2-qRT-F	AGCAGATTGGATGTAATGGCGATTCC	
GA2ox2-qRT-R	TCTCACACGCCATTTTCTTCACTGC	
GA2ox3-qRT-F	TCAGCAATGAAGAAAATGGCATGTGA	
GA2ox3-qRT-R	GCACCAAATCCAATCAAATTTCTGCCA	
GA2ox4-qRT-F	AAAGTCGTCAACCATAGCGTCCCTA	
GA2ox4-qRT-R	TTCGACCCAACCCAAATCACCATTG	
GA3ox2-qRT-F	GATGGTATTGCTGGTTATGGCGTGG	
GA3ox2-qRT-R	CCACATCAGTCTTCCGGCTAGTTTT	
GA20ox1-qRT-F	GCGTAGGGAGGGAACACTTCAAAGA	
GA20ox1-qRT-R	AAACTTGAAGCCCGCCAACACTATC	
GA20ox2-qRT-F	AGGCAATGAAGAGGGTAGGACTTGT	
GA20ox2-qRT-R	GTGCAGTTCCAAGGGCTAAATCAGG	
GA20ox3-qRT-F	CCCTTGAGGCTTGGACAAATGGAAG	
GA20ox3-qRT-R	AGATGCCTCAGTTCAGCCCATGTAA	
ZmActin-F	GTTGGGCGTCCTCGTCA	
ZmActin-R	TGGGTCATCTTCTCCCTGTT	
NtActin-F	GAGCGGGAAATTGTCAGGGA	
NtActin-R	GAAACGCTCAGCACCAATGG	

### Gene Isolation and Phylogenetic Analysis

*ZmDREB4.1* was isolated from maize roots with primer pair ZmDREB4.1-F/R (**Table 1**), and the PCR product was then cloned into the pMD19T vector. The amino acid sequences of ZmDREB4.1 and other DREB A-4 subgroup transcription factors in this study were obtained from Phytozome^1^ or NCBI^2^. Multiple sequence alignment was performed using ClustalX software, and the phylogenetic tree was generated using MEGA5.1 software.

### Subcellular Localization

The CDS sequence of *ZmDREB4.1* without termination codon was cloned into the 5′ terminus of the enhanced green fluorescent protein (eGFP) gene sequence in pUC-eGFP vector via *Xba*I/*Sma*I sites (primer pair: ZmDREB4.1-SL-F/R; **Table 1**). Protoplasts of etiolated maize mesophyll cells were isolated using macerozyme R-10 and cellulase R-10 (Yakult, Japan) and the fusion construct pUC-ZmDREB4.1-eGFP and the control pUC-eGFP were transformed into the protoplasts independently using PEG-Ca^2+^ method according to the protocol described by Sheen (Sheen, 2002). After culture in W1 solution (0.6 M mannitol, 4 mM MES (pH 5.7), 4 mM KCl) in the dark for 14 h, the green fluorescence signals were observed under a confocal microscope (Zeiss 510, Carl Zeiss, Germany). The young stems were cut into thin slices and then treated on NB medium containing 0.4 M mannitol for 4–6 h. The plasmids mentioned above were transformed into young stem tissue independently by particle bombardment with the PDS-1000 system (Bio-Rad, United States) according to the protocol reported by Wang et al. (2006). GFP fluorescence was observed after further culture overnight.

### Yeast One-Hybrid Assay

The 3 × DRE sequence (DRE: GATATACTACCGACATGAGTTC, between positions -174 and -153 in the *rd29A* promoter; **Table 1**) and 3 × mDRE sequence (The DRE core sequence ACCGAC was substituted with AAAAAA; **Table 1**) were inserted into the pAbAi vector via the *Hin*dIII/*Xho*I sites to create the bait and mutant bait vectors. The vectors were transformed into yeast strain Y1HGold independently and they were then screened on SD/-Ura medium. The positive yeast was grown on SD/-Ura medium which contains Aureobasidin A (AbA) at different concentrations to examine activity of self activation according to the protocol of the Matchmaker^TM^ gold Yeast One-Hybrid Library Screening System (PT4087-1). The CDS sequence of *ZmDREB4.1* was constructed into the pGADT7-AD vector via *Eco*RI/*Bam*HI sites as prey vector (Primer pair: ZmDREB4.1-YH-F/R; **Table 1**). It was transformed into bait and mutant bait strains, respectively, and then grown on SD/-Leu medium containing 800 ng mL^-1^ AbA for 3 days at 30°C.

### Transcriptional Activation Assay in Yeast

The CDS of *ZmDREB4.1* was cloned into the 3′ terminus of the GAL4 binding domain sequence in pBD-GAL4 vector via *Eco*RI/*Sal*I sites (Primer pair: ZmDREB4.1-TA-F/R; **Table 1**). The plasmids pBD-ZmDREB4.1, pGAL4 (positive control) and pBD-GAL4 (negative control) were independently transformed into yeast strain YRG-2 which contains the *HIS3* and *lacZ* reporter genes. These transformed yeast cells were grown on SD/-Trp or SD/-Trp/-His medium for 3 days at 30°C. The β-galactosidase activity of the transformed yeast cells was also analyzed according to the protocol described in the Yeast Protocols Handbook (PT3024-1).

### Transcriptional Activation Assay in Tobacco

For the transcriptional activation assay of ZmDREB4.1 in tobacco, the full-length CDS of *ZmDREB4.1* was inserted into the pCambia1300 vector to construct the effector plasmid (Primer pair: ZmDREB4.1-ORF-F/R; **Table 1**). The reporter plasmid was constructed by placing the cascade of 3 × DRE and mini 35S upstream of the GUS reporter gene in pCambia1381 (Primer pair: mini35S-F/R; **Table 1**). The effector plasmid (pSuper::ZmDREB4.1), reporter plasmid (p3 × DRE:mini35S::GUS), internal control plasmid (p35S::LUC), enhancer plasmid (p19) and control plasmid (pCambia1300) were transformed into *Agrobacterium tumefaciens* strain GV3101 independently. The tobacco seedlings were grown at 23°C under a long-day photoperiod (16 h light, 8 h dark) in a greenhouse for 4–5 weeks until the six-leaf stage. Three separate tobacco leaves were infected with the mixed *Agrobacterium* solution (pSuper::ZmDREB4.1: p3 × DRE:mini35S::GUS: p35S::LUC:p19 = 800:100:4:300) as three biological replicates. The mixed solution in which pSuper::ZmDREB4.1 was substituted with pCambia1300 served as the control. After inoculation for 3 days, total protein was extracted from these transformed leaves to measure GUS and LUC activities. The LUC activities were quantified using the Luciferase Assay System (Promega, United States) and the GUS activities were measured by spectrofluorometry (F-4500, HITACHI, Japan). The relative GUS/LUC activity was used to represent promoter activity. The column diagram was generated by GraphPad Prism software using the results from three biological replicates and the statistically significant differences were evaluated by Student’s *t*-test (^∗^*P*-value < 0.05, ^∗∗^*P*-value < 0.01, ^∗∗∗^*P*-value < 0.001).

### Electrophoretic Mobility Shift Assay (EMSA)

The full-length ZmDREB4.1 protein fused with 6 × His tag, GST tag or MBP tag was not obtained in the prokaryotic expression system. Therefore, the truncated sequence containing the coding sequence of the conserved AP2 domain of ZmDREB4.1 (31-147 amino acids) was cloned into the pMAL-C2x vector via *Eco*RI/*Bam*HI sites (Primer pair: ZmDREB4.1-PE-F/R; **Table 1**) and expressed in *Escherichia coli* strain TB1. The recombinant protein was purified using MBP Sepharose High Performance (GE Healthcare, United States) following the manufacturer’s protocol and then used for the EMSA. The probe (3 × DRE labeled with biotin at the 5′ terminus), the competitor1 (3 × DRE) and the competitor2 (3 × mDRE) were synthesized (Sangon Biotech, China). The double-stranded probes were obtained by incubating at 95°C for 5 min and cooling gradually overnight. The gel-shift assay was carried out using the LightShift^®^ Chemiluminescent EMSA Kit (Thermo, United States) following the manufacturer’s instructions.

### Generation of Transgenic Tobacco Plants

The full-length CDS of *ZmDREB4.1* was amplified with primer pairs ZmDREB4.1-ORF-F/R (**Table 1**) and then constructed into the binary vector pBI121. The recombinant vector pBI121-ZmDREB4.1 was transformed into *A. tumefaciens* strain LBA4404. The positive clone was used for tobacco transformation with the leaf disk method. The transformed leaves were cultured on MS medium in the dark for 3 days and then transferred to differentiation medium (MS medium containing 0.2 mg L^-1^ NAA, 3 mg L^-1^ 6-BA and 50 mg L^-1^ kanamycin) under the light. The mediums were replaced every 2 weeks. The regenerated buds rooted on MS medium to generate T0 plants. PCR amplification was used to confirm the positive lines with primer pair ZmDREB.41-F/R (**Table 1**). The expression levels of *ZmDREB4.1* in transgenic lines were examined by qRT-PCR using primer pair ZmDREB4.1-qRT-F/R (**Table 1**).

### Phenotype Observation and Measurement of Transgenic Tobacco

The sterile seeds of WT and *ZmDREB4.1*-overexpression lines were sown on MS medium. The WT and transgenic seedlings at the two-leaf stage were chosen for hypocotyl observation and length measurement. The length of the stems and second petioles and the area of the second leaves of seedlings at the four-leaf stage were observed and measured. The data were collected from 25 seedlings of each of three independent transgenic lines. A scanning electron microscope (SEM) was used for fine structure observation (Olympus 6000, Japan). The hypocotyls and petioles were consistently observed in the mid-region. For leaves, adaxial epidermis was observed. The numbers of leaf epidermal cells and hairs were counted from 10 leaves. For measurements of cell length of the hypocotyl and petiole, data was collected from at least 40 cells of two different hypocotyls and petioles. The column diagram was generated by GraphPad Prism software and the statistically significant differences were evaluated by Student’s *t*-test (^∗^*P*-value < 0.05, ^∗∗^*P*-value < 0.01, ^∗∗∗^*P*-value < 0.001) and corrected by Bonferroni correction.

### Analysis of Callus Formation

The top leaves and second full-length petioles of seedlings at the four-leaf stage were obtained. The leaves were cut into small pieces of similar size. About 60 pieces from 30 separate plants were precultured on four callus-induced medium (CIM, MS medium containing 1 mg L^-1^ 2,4-D, and 0.2 mg L^-1^ 6-BA) for 4 days. Also, about 20 full-length petioles from 20 separate seedlings were precultured on two CIM for 4 days. They were then transferred to new CIM for further culture for 2 weeks. The calli induced from leaves and petioles were observed and the calli induced from leaves were measured. The column diagram was generated by GraphPad Prism software using the fresh weight of the 60 calli and the statistically significant differences were evaluated by Student’s *t*-test (^∗^*P*-value < 0.05, ^∗∗^*P*-value < 0.01, ^∗∗∗^*P*-value < 0.001). The protocol was reported by Mehrnia et al. (2013).

### Measurement of Endogenous Phytohormone Levels

The sterile seeds of WT and *ZmDREB4.1*-overexpression tobacco were grown on MS medium at 28°C for 10–14 days until the four-leaf stage. The leaves and petioles of WT and OE9 were obtained. The leaves and petioles were divided into three parts, respectively, and the fresh weight of every part was about 0.2 g. Then they were frozen in liquid nitrogen and homogenized to a fine powder. The powder was immersed in 3 mL of 80% methanol for 3 h and then extracted with another 1 mL of 80% methanol for 1 h. The IAA, ZR, DHZR, IPA, GA3, and GA4 contents were measured with their monoclonal antibodies using the indirect competitive enzyme-linked immunosorbent assay (icELISA) as described by Deng et al. (2008). The column diagrams were generated by GraphPad Prism software using the results from three biological replicates and the statistically significant differences were evaluated by Student’s *t*-test (^∗^*P*-value < 0.05, ^∗∗^*P*-value < 0.01, ^∗∗∗^*P*-value < 0.001).

### Hormonal Treatment

The WT and *ZmDREB4.1*-overexpressing tobacco seedlings were grown on MS medium for 5 days. The WT seedlings were transplanted into new MS medium. The transgenic seedlings were transplanted into new MS medium or medium containing GA3, NAA, or 6-BA at different concentrations. The hypocotyl, stem and petiole length and leaf size of WT and transgenic seedlings grown on different media was observed and measured after culture for 2 weeks. The data was collected from 15 separate seedlings of WT and OE9. The column diagrams were generated by GraphPad Prism software and multiple means were compared using Tukey’s multiple comparison test (α = 0.05).

### Callus Transformation in Maize

The *bar* gene expression cassette was inserted into the pUC-ZmDREB4.1-eGFP and pUC-eGFP vector via *Hin*dIII site to construct the overexpression and control vectors. The calli were treated on NB medium containing 0.4 M mannitol for 4–6 h. These two vectors were transformed into treated calli by particle bombardment independently. The bombarded calli were further cultured overnight and transferred to NB medium containing 0.2 mg L^-1^ 2,4-D for 2 weeks. A fluorescence microscope was used to examine GFP fluorescence. The calli with green fluorescence were observed and measured. Then, they were cultured on selection medium [NB medium containing 0.2 mg L^-1^ 2,4-D and 3 mg L^-1^ bialaphos (Kyocera, Japan)] for 2 months. The medium was replaced every 2 weeks. Subsequently, the resistant calli were recovered on NB medium containing 50 g L^-1^ sucrose for 1 week and then transferred to differentiation medium to regenerate seedlings.

### Accession Numbers

The GenBank accession number of each gene in this study is as follows: *ZmDREB4.1* (GRMZM2G028969), *DBF2* (GRMZM5G889719), *TINY* (AT5G25810), *TINY2* (AT5G11590), *GhDBP3* (DQ224382), *StDREB1* (JN125862), *MnDREB4A* (KF678389), *YUCCA9* (XM_016635159), *YUCCA10* (XM_016642753), *YUCCA11* (XM_016649400), *IPT3* (XM_016647510), *IPT5* (XM_016584942), *GA2ox2* (AB125233), *GA2ox3* (EF471117), *GA2ox4* (KC568201), *GA3ox2* (EF471116), *GA20ox1* (NM_001326160), *GA20ox2* (XM_016615759), and *GA20ox3* (XM_016648485).

## Results

### Isolation and Expression Analysis of *ZmDREB4.1*

**FIGURE 1:**
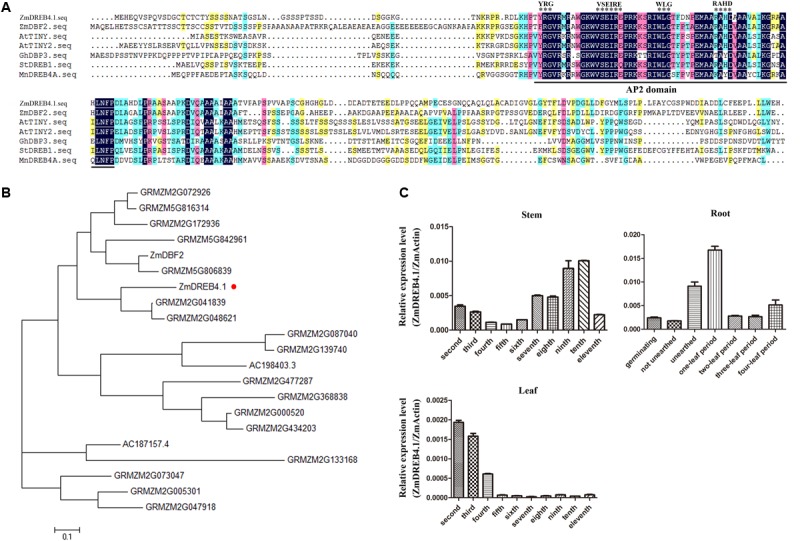
Multiple sequence alignment and expression analysis of *ZmDREB4.1*. **(A)** Multiple sequence alignment between ZmDREB4.1 and several reported DREB A-4 subgroup members from various species. The conserved AP2 domain is marked by a black line and the VSEIRE/Q, YRG, RAYD, and WLG motifs are marked by black asterisks. **(B)** Phylogenetic analysis of the DREB A-4 subgroup transcription factors from maize. The tree was generated by the neighbor-joining method using the full-length protein sequences. ZmDREB4.1 is highlighted by a round red dot. **(C)** The relevant expression levels of *ZmDREB4.1* (the expression level of *ZmDREB4.1*/the expression level of *ZmActin*) analyzed by qRT-PCR in maize roots, stems and leaves.

*ZmDREB4.1* was cloned by PCR from maize root cDNA. The *ZmDREB4.1* gene contains an open reading frame of 759 bp encoding a protein composed of 252 amino acid residues. It is reported that the transcription factors of DREB subfamily possess a conserved AP2 DNA-binding domain and YRG, WLG, and RAHD motifs (Okamuro et al., 1997). The DREB transcription factors were divided into six subgroups according to the sequence specificity localized from the 14th to 19th residues of the AP2 DNA-binding domain (Sakuma et al., 2002). *DBF2* (Kizis and Pagès, 2002), *TINY* (Wilson et al., 1996), *TINY2* (Wei et al., 2005), *GhDBP3* (Huang and Liu, 2006), *MnDREB4A* (Liu et al., 2015), and *StDREB1* (Bouaziz et al., 2013) belonging to DREB A-4 subgroup have been reported. The sequence alignment between ZmDREB4.1 and these transcription factors was performed. As shown in **Figure 1A**, ZmDREB4.1 had a conserved AP2 domain; YRG, WLG, and RAHD motifs; and VSEIRE sequence at the 14th to 19th position, which was consistent with these known DREB A-4 subgroup transcription factors. The results indicated that *ZmDREB4.1* was classified into the A-4 subgroup of the DREB subfamily.

Furthermore, 21 DREB A-4 subgroup transcription factors were identified from maize according to the conserved AP2 DNA-binding domain and VSEIRE/Q sequence. A phylogenetic tree was generated using amino acid sequences of these 21 proteins by the MEGA program and the neighbor-joining method (**Figure 1B**). *DBF2*, a DREB A-4 subgroup gene from maize, has been reported to play a negative role in the regulation of the promoter activity of *rab17* (Kizis and Pagès, 2002). Phylogenetic tree analysis placed ZmDREB4.1 and DBF2 into two different branches. The AP2 DNA-binding domains of ZmDREB4.1 and DBF2 shared 89.66% amino acid identity. They shared low amino acid identity outside of the AP2 DNA-binding domain.

The expression levels of *ZmDREB4.1* in different maize tissues were analyzed using qRT-PCR. The roots at germinating, not unearthed, unearthed, one-leaf, two-leaf, three-leaf, and four-leaf stage and second to eleventh stems and leaves of V11-stage maize were selected. The results showed that *ZmDREB4.1* was expressed in roots of different stages and that the expression of *ZmDREB4.1* peaked at the V1 stage. *ZmDREB4.1* was also expressed in the stems and leaves of V11-stage maize. The expression of *ZmDREB4.1* decreased gradually from the second to fifth stems and then rose from the sixth to tenth stems. Transcript levels of *ZmDREB4.1* decreased from the second to fourth leaves and were almost not examined from the fifth to eleventh leaves (**Figure 1C**). It is reported that the expression levels of most DREB genes are induced by various abiotic stresses (Agarwal et al., 2017). We examined the expression levels of *ZmDREB4.1* under various abiotic and biotic stresses. Three-leaf seedlings were treated with 20% PEG6000, 100 mM NaCl, cold (4°C), wound, 1 μM NAA, 10 μM 6-BA, 10 μM GA3, or 10 μM ACC. *ZmDREB4.1* expression was not induced by any of the above treatments (Supplementary Figure S1).

### ZmDREB4.1 Is Located in the Nucleus and Functions as a Transcriptional Activator

**FIGURE 2:**
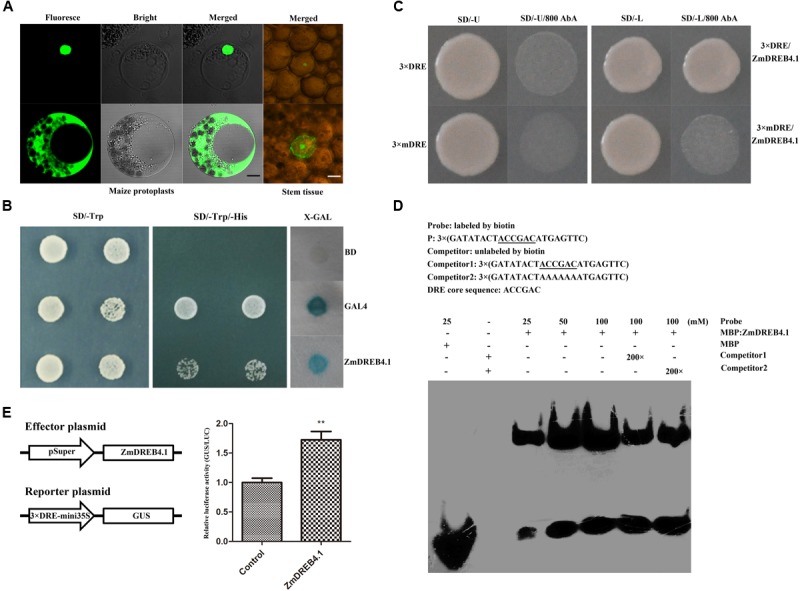
Transcription factor characteristics of ZmDREB4.1. **(A)** Subcellular localization of the ZmDREB4.1 transcription factor in maize mesophyll protoplasts and young stem tissue. The black and white scale bars indicate 5 and 10 μm, respectively. **(B)** Transcriptional activation analysis of ZmDREB4.1 in the yeast. GAL4 and BD indicate the positive control and negative control, respectively. **(C)** Yeast one-hybrid assay. ZmDREB4.1 binds to the DRE element, but not the mDRE element. **(D)** EMSA. The DRE core sequence is marked by underlines. ZmDREB4.1 specifically binds to the DRE element. **(E)** Transcriptional activation analysis of ZmDREB4.1 in tobacco leaves. GUS/LUC value indicated ZmDREB4.1 activates the expression of GUS gene driven by the fusion promoter of 3 × DRE and mini 35S. The leaves co-transformed with the empty p1300Super effector, the GUS reporter, p19 and p35S::LUC severed as the control. The GUS/LUC value of the control was set to 1. The values represent the mean ± SD from three biological replicates and the statistically significant differences were evaluated by Student’s *t*-test (^∗∗^*P*-value < 0.01).

We examined the subcellular localization of ZmDREB4.1. The *ZmDREB4.1-GFP* fusion gene, under the control of the CaMV 35S promoter, was transiently expressed in maize mesophyll protoplasts and young stem tissue. The p35S::GFP plasmid was used as a control and transfected independently. As shown in **Figure 2A**, the green fluorescent signals accumulated only in the nucleus of cells transfected with ZmDREB4.1-GFP, while the signals were observed in both the nucleus and cytoplasm of the control. These results confirmed that ZmDREB4.1 was located in the nucleus.

According to previous reports, the members of the DREB subfamily can bind to the DRE element (Yamaguchi-Shinozaki and Shinozaki, 1994). The yeast one-hybrid system and EMSA were used to determine the binding characteristics of ZmDREB4.1 to the DRE element. The bait (pABAi-3 × DRE) and mutant bait (pABAi-3 × mDRE) plasmids were transformed into yeast strain Y1HGold independently to generate bait and mutant bait strains. The bait stain transfected with prey vector (pGADT7-ZmDREB4.1) could grow on SD/-Leu medium containing 800 ng ml^-1^ AbA, while mutant bait stain could not (**Figure 2C**). For EMSA, the MBP-ZmDREB4.1_31-147__aa_ fusion protein containing the AP2 DNA-binding domain was obtained by the prokaryotic expression system (Supplementary Figure S2). The 3 × DRE oligonucleotide was labeled with biotin at the 5′ terminus and used as the probe. The retardant band indicated that ZmDREB4.1_31-147__aa_ could bind to the DRE element directly (**Figure 2D**).

Yeast and tobacco assay systems were used to determine whether ZmDREB4.1 was able to activate transcription. The pBD-ZmDREB4.1, pGAL4 (positive control) and pBD-GAL4 (negative control) plasmids were transfected into yeast strain YRG-2 individually. The yeast transfected with pBD-ZmDREB4.1 and pGAL4 could grow on the SD/-Trp/-His, but the negative control could not. In addition, blue color was observed when the extract of yeast transfected with pBD-ZmDREB4.1 and pGAL4 was incubated with *X*-gal (**Figure 2B**). In tobacco leaves, the relative GUS/LUC activity of the extract of leaves transfected with pSuper::ZmDREB4.1 and p3 × DRE:mini35S::GUS was approximately 1.7 times as high as the control (**Figure 2E**), indicating that ZmDREB4.1 promoted the activity of the p3 × DRE:mini35S promoter. These results demonstrated that ZmDREB4.1 could bind to the DRE element directly and functioned as a transcriptional activator.

### The Differences Between Transgenic Lines and WT in Tobacco

*ZmDREB4.1* was overexpressed in tobacco to investigate the function of *ZmDREB4.1*. The transgenic tobacco plants were examined by PCR, and 19 transgenic lines were obtained. The expression levels of *ZmDREB4.1* were analyzed by qRT-PCR. Three lines (OE6, OE9 and OE13) with high expression levels were selected for further investigations (Supplementary Figure S3).

**FIGURE 3:**
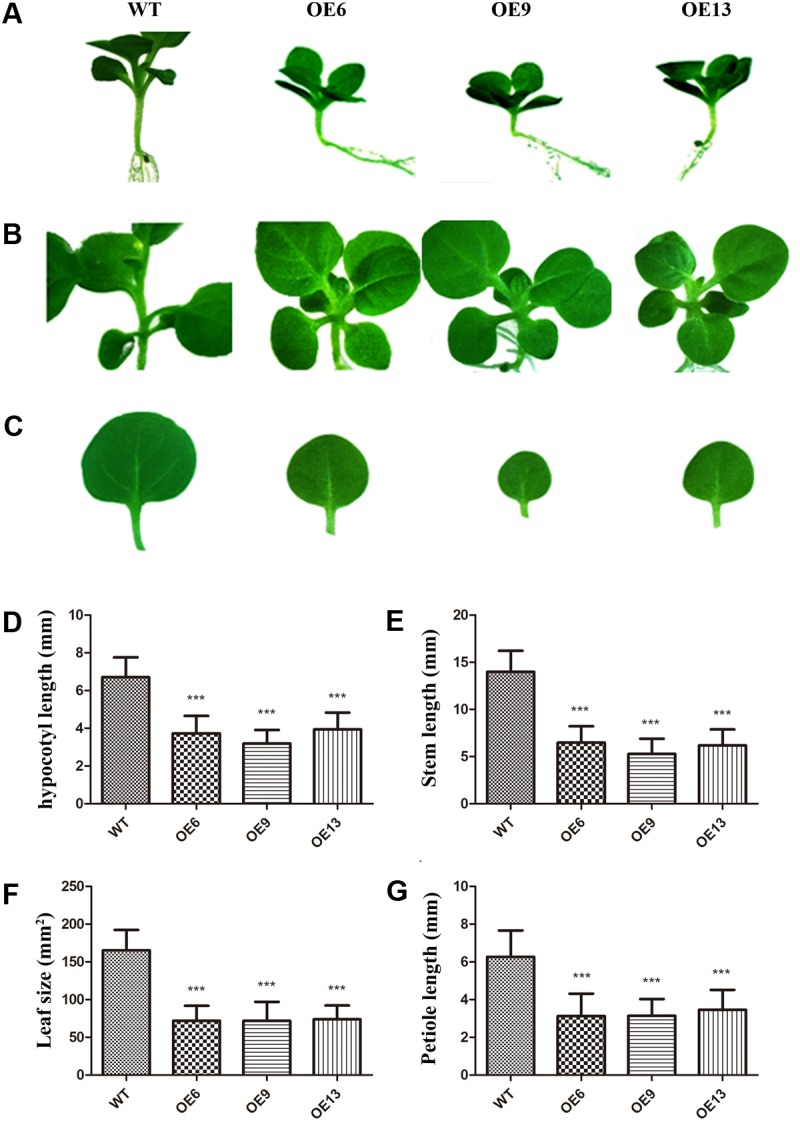
Phenotypes of *ZmDREB4.1*-overexpressing tobacco. Compared with WT, *ZmDREB4.1-*overexpressing tobacco exhibited shorter hypocotyls **(A)**, stems **(B)** and petioles and smaller leaves **(C)**. The tobacco plants at the two-leaf stage were chosen for hypocotyl observation, and tobacco plants at the four-leaf stage were chosen for observation of stem, petiole, and leaf. Scale bar: 5 mm. Column diagrams in **(D–G)** showed quantitative determination of hypocotyl length, plant height, petiole length and leaf size, respectively. For **(D–G)**, the values represent the mean ± SD from 25 separate plants of each of three independent transgenic lines and the statistically significant differences were evaluated by Student’s *t*-test and corrected by Bonferroni correction (^∗∗∗^*P*-value < 0.001/3).

The transgenic and WT plants were grown on MS medium at 28°C under a long-day photoperiod (16 h light, 8 h dark) to explore whether there were some phenotypes in *ZmDREB4.1*-overexpressing lines. Compared with WT, the overexpression of *ZmDREB4.1* gave rise to obvious changes in morphology. *ZmDREB4.1*-overexpressing plants exhibited a dwarf phenotype with shorter hypocotyls, stems and petioles and smaller leaves (**Figures 3A–C**). The average hypocotyl length of OE6, OE9, and OE13 at the two-leaf stage was 3.7, 3.2, and 3.9 mm which were 55.58, 47.51, and 58.71% as long as the control (6.7 mm) (**Figure 3D**). The average stem length of OE6, OE9, and OE13 at the four-leaf stage was 0.65, 0.53, and 0.62 cm which were significantly shorter than the control (1.4 cm) (**Figure 3E**). The second petiole lengths of OE6, OE9, and OE13 at the four-leaf stage (3.12, 3.14, and 3.47 mm) were 49.89, 50.09 and 55.35% as long as the control (6.26 mm) (**Figure 3G**). The areas of the second leaves of OE6, OE9, and OE13 (0.72, 0.72, and 0.74 cm^2^) at the four-leaf stage were significantly smaller than those of the control (1.65 cm2) (**Figure 3F**). However, the number of the leaves of OE6, OE9, or OE13 was not influenced during the entire life cycle (Supplementary Figure S4). In addition, there were some differences at reproductive stage such as the shorter pedicel and earlier abscission of flowers (Supplementary Figure S5). We also examined *ZmDREB4.1*-overexpressing tobacco tolerance to drought. The transgenic plants displayed reduced drought tolerance compared with WT after they were subjected to drought treatment for 2 weeks (Supplementary Figure S6). These results demonstrated that overexpression of *ZmDREB4.1* repressed plant growth and development and resulted in reduced drought tolerance in tobacco.

### Overexpression of *ZmDREB4.1* Repressed Cell Division in Transgenic Leaves

**FIGURE 4:**
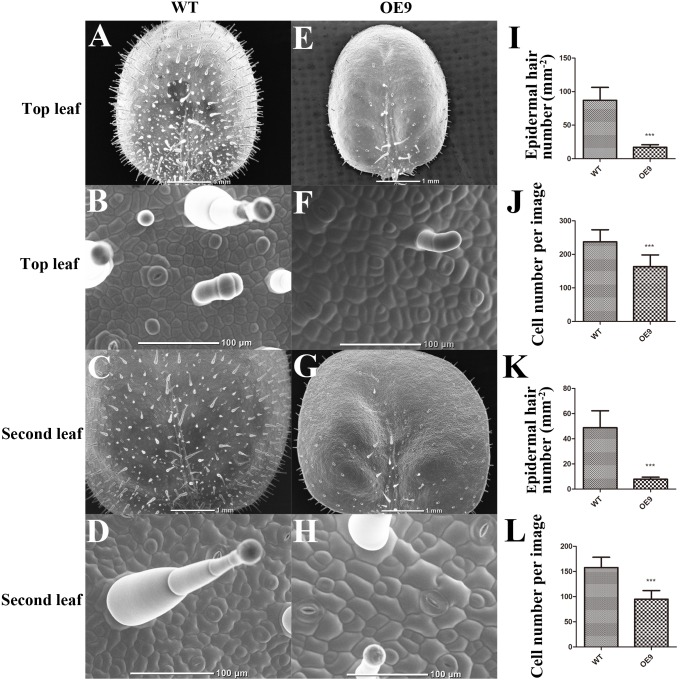
Scanning electron microscope (SEM) observation and calculation of the leaves. SEM observation of adaxial leaves of WT and OE9. **(A,E)** Top leaf. **(C,G)** Second leaf. **(B,F)** The enlargement of **(A,E)**, respectively. **(D,H)** The enlargement of **(C,G)**. The epidermal hair number per mm^2^ of the top **(I)** and second leaves **(K)** and the average cell number per image of the top **(J)** and second leaves **(L)** were calculated. Scale bar: 1 mm in **(A,C,E,G)** and 100 μm in **(B,D,F,H)**. For **(I–L)**, the values represent the mean ± SD from 10 separate plants of WT and OE9 and the statistically significant differences were evaluated by Student’s *t*-test (^∗∗∗^*P*-value < 0.001).

Fewer cells or smaller cells can give rise to smaller leaves. To determine the exact reason, a SEM was used to observe the structure and cell size of OE9 and WT. The cell sizes of the top and second leaves of OE9 seedlings (**Figures 4F,H**) at the four-leaf stage were larger than those of the WT (**Figures 4B,D**). There was approximately 163 and 237 cells per image in the top leaves of OE9 and WT, respectively (**Figure 4J**), and the numbers of cells were 95 and 158 in the second leaves (**Figure 4L**). In addition, we observed that the number of epidermal hairs of OE9 seedlings (**Figures 4E,G**) was significantly lower than that of WT (**Figures 4A,C**). The average numbers of epidermal hairs of OE9 were approximately 19.54 and 15.98% as great as those of WT in the top and second leaves, respectively (**Figures 4I,K**). The initiation of epidermal hairs relies on reactivation of endoreduplication (Payne et al., 1999). The fewer cells and epidermal hairs in OE9 indicated that overexpression of *ZmDREB4.1* repressed cell proliferation in transgenic leaves.

### *ZmDREB4.1* Overexpression Repressed Callus Growth in Tobacco

**FIGURE 5:**
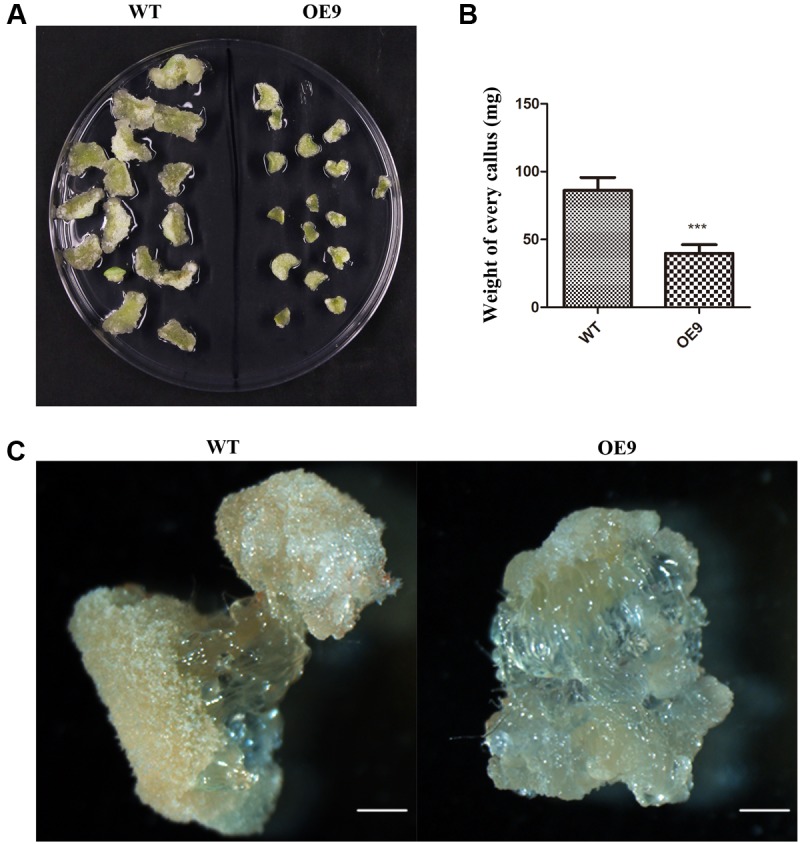
Callus-induction assay of tobacco leaf and petiole. **(A)** Morphology of calli induced from top leaf of the four-leaf-stage plants after culture for 2 weeks. **(B)** The average fresh weight of calli in **(A)**. The values represent the mean ± SD from 60 calli and the statistically significant differences were evaluated by Student’s *t*-test (^∗∗∗^*P*-value < 0.001). **(C)** The calli induced from second petioles of the four-leaf-stage plants after culture for 2 weeks. OE9 showed smaller callus. Scale bar: 1 mm in **(C)**.

To further analyze the function of *ZmDREB4.1* in cell proliferation, a callus-induction assay was performed. The top leaves of WT and OE9 seedlings at the four-leaf stage were cut into small pieces of similar size. After being cultured for 2 weeks, the calli induced from leaves of OE9 grew slowly and were significantly smaller than those of WT (**Figure 5A**). The results from the calli cultured on four CIM were coincident. Accordingly, the fresh weight of calli induced from OE9 leaves was on average 39.81 mg, while this value was 86.22 mg in WT (**Figure 5B**). Similarly, the second petioles from 20 separate plants of OE9 at four-leaf stage generated fewer calli compared with WT (**Figure 5C**). These results indicated that overexpression of *ZmDREB4.1* inhibited cell proliferation during callus induction in tobacco.

### Shorter Hypocotyls and Petioles Were Because of Inhibition of Cell Elongation

**FIGURE 6:**
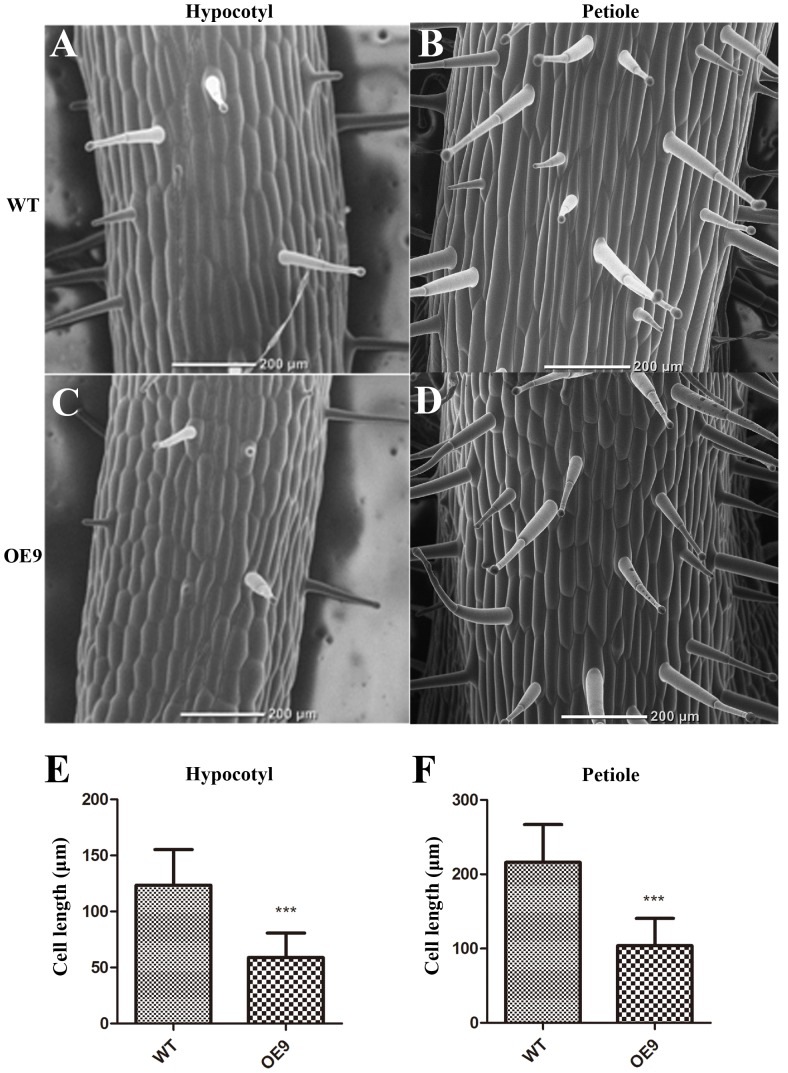
Scanning electron microscope observation and the statistics of cell length of the hypocotyls and petioles. **(A,C)** Indicated the cell morphology of hypocotyls of the two-leaf-stage plants. **(B,D)** Indicated cell morphology of the second petioles of the four-leaf-stage plants. The average cell length of the hypocotyls **(E)** and petioles **(F)** were calculated. OE9 possessed shorter cells in both hypocotyl and petiole. For **(E)** and **(F)**, the values represent the mean ± SD of the data from at least 40 cells of two different hypocotyls and petioles and the statistically significant differences were evaluated by Student’s *t*-test (^∗∗∗^*P*-value < 0.001).

We also examined why the length of hypocotyls and petioles of OE9 became shorter. The hypocotyls at the two-leaf stage and second petioles at the four-leaf stage of OE9 and WT were selected to be observed. The cell sizes of OE9 hypocotyls (**Figure 6C**) were shorter than those of WT (**Figure 6A**). The average cell length of OE9 was 58.98 μm, while this value of WT was 123.48 μm (**Figure 6E**). Similar results were observed in petioles of OE9 (**Figure 6D**) and WT (**Figure 6B**). The average cell length of OE9 was approximately 48.03% as long as that of WT (**Figure 6F**). The hypocotyl and petiole length in OE9 decreased by 52.49% (**Figure 3D**) and 49.91% (**Figure 3G**), respectively, which was close to the reduced degree of cell length of the hypocotyl and petiole (52.24 and 51.97%). These results indicated that the shorter hypocotyls and petioles of OE9 resulted from inhibition of cell elongation rather than cell division.

### Overexpression of *ZmDREB4.1* Decreased Auxin, CK and GA Levels and Application of GA3 Rescued the Dwarf Phenotype in Transgenic Tobacco

**FIGURE 7:**
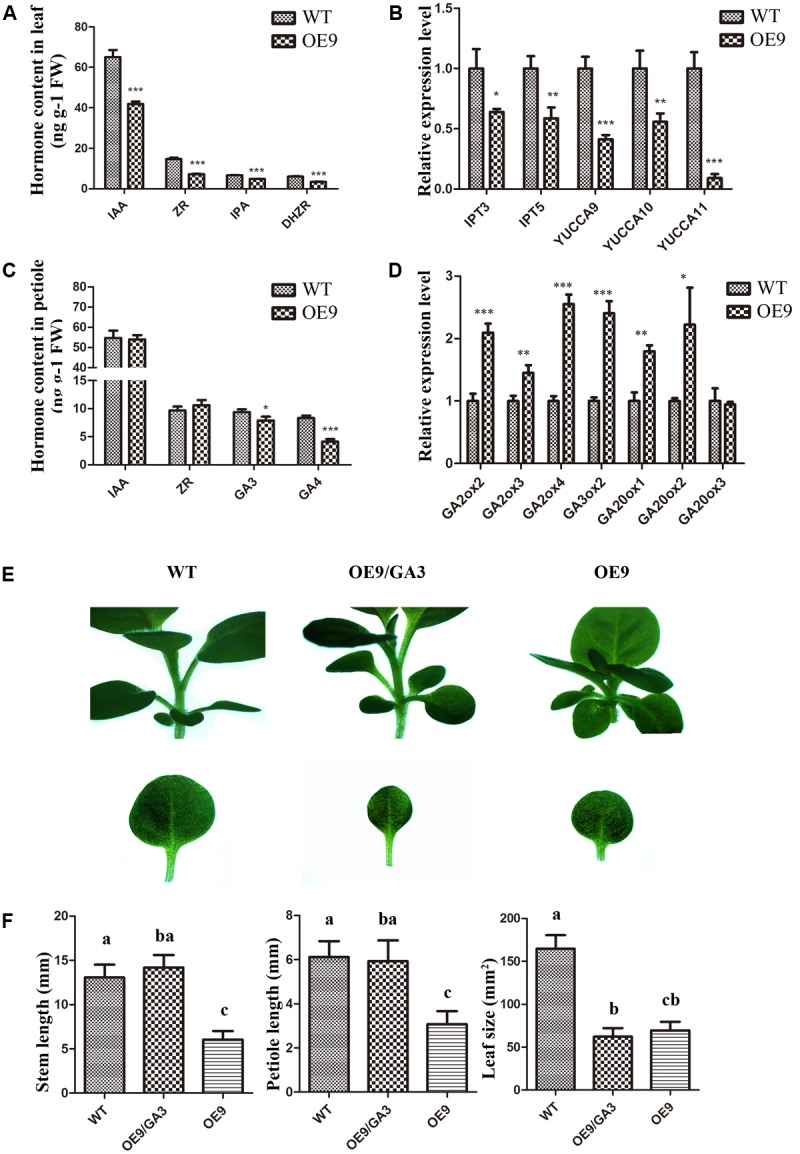
Analysis of the endogenous phytohormone levels and the expression levels of relative genes and effects of GA3 on phenotypic restoration. **(A)** IAA, ZR, DHZR, and IPA contents in WT and OE9 leaves. **(B)** The expression levels of *YUCCA9*, *YUCCA10,* and *YUCCA11* which were involved in the biosynthesis of auxin and *IPT3* and *IPT5* which were involved in the biosynthesis of cytokinin. **(C)** GA3 and GA4 contents in WT and OE9 petioles. **(D)** The expression levels of *GA2ox2*, *GA2ox3,* and *GA2ox4* which were involved in the biosynthesis of GA, and *GA3ox2*, *GA20ox1*, *GA20ox2,* and *GA20ox3* which were involved in conversion to inactive forms in GA metabolism. For **(A–D)**, the values represent the mean ± SD of the results from three biological replicates and the statistically significant differences were evaluated by Student’s *t*-test (^∗^*P*-value < 0.05, ^∗∗^*P*-value < 0.01, ^∗∗∗^*P*-value < 0.001). **(E)** The shorter-hypocotyl, -stem and -petiole phenotypes were rescued by application of exogenous GA3 at 1 μM. Scale bar: 5 mm. **(F)** The stem and petiole length and leaf size of WT, OE9 and OE9 cultured on CIM containing 1 μM GA3. The valves represent the mean ± SD from 15 separate plants of WT and OE9 and multiple means were compared using Tukey’s multiple comparison test (α = 0.05). The letters (a,b,c) represent the significant difference.

Some mutants deficient in phytohormone biosynthesis and response exhibit dwarfism (Choe et al., 1998; Talon et al., 1999; Griffiths et al., 2006) which resembles the phenotype of *ZmDREB4.1*-overexpressing tobacco. This reminded us to pay attention to the change in endogenous phytohormone levels. We obtained the leaves and petioles of OE9 and WT. They were divided into three parts and used to measure endogenous phytohormone levels by icELISA. Compared with WT, the IAA, ZR, DHZR, and IPA contents were reduced by 35.52, 50.55, 42.60, and 28.51% in OE9 leaves (**Figure 7A**). Further analysis showed that the expression levels of *YUCs* (*YUCCA9*, *YUCCA10,* and *YUCCA11*) involved in biosynthesis of auxin (Stepanova et al., 2011) and *IPTs* (*IPT3* and *IPT5*) involved in biosynthesis of CK (Lindner et al., 2014) were significantly down-regulated in OE9 leaves as well (**Figure 7B**). In OE9 petioles, the GA3 and GA4 contents were reduced by 15.75 and 50.47%, while the IAA and ZR contents were not significantly different from WT (**Figure 7C**). The *GA2ox* genes (*GA2ox2*, *GA2ox3,* and *GA2ox4*) involved in the conversion of bioactive GAs to inactive forms in GA metabolism (Thomas et al., 1999) were obviously up-regulated in OE9. However, the genes (*GA3ox2*, *GA20ox1*, and *GA20ox2*) involved in biosynthesis of bioactive GA (Xu et al., 1995) were also up-regulated (**Figure 7D**). Therefore, we proposed that the reduced bioactive GA levels in OE9 were because of up-regulation of *GA2ox* genes, whereas up-regulation of *GA20ox* and *GA30ox* genes might result from feedback mechanism in GA homeostasis regulation.

Since *ZmDREB4.1*-overexpressing tobacco contained reduced auxin, CK and GA contents, we investigated whether exogenous hormone could rescue phenotypes in *ZmDREB4.1*-overexpressing tobacco. The OE9 and WT seedlings were grown on MS medium or MS medium containing NAA, 6-BA or GA3 at different concentrations for 2 weeks. The results showed that the shorter stems and petioles were rescued by 1 μM GA3 (**Figure 7E**). The stem and petiole length of OE9 when they were cultured on the medium containing GA3 was close to those of WT cultured on MS medium, respectively (**Figure 7F**). However, the exogenous GA3 failed to rescue the smaller-leaf phenotype in OE9 (**Figures 7E,F**). In addition, we did not observe recovery of the smaller-leaf phenotype by NAA or 6-BA. These results demonstrated that the dwarf phenotype of *ZmDREB4.1*-overexpressing tobacco might result from deficiency of GA.

### Overexpression of *ZmDREB4.1* Repressed Callus Growth and Regeneration in Maize

**FIGURE 8:**
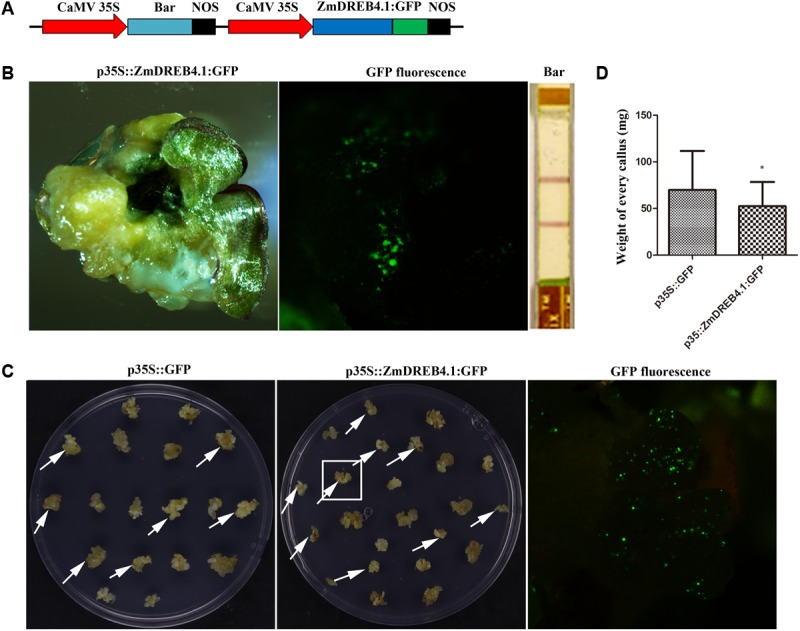
*ZmDREB4.1* repressed callus growth and regeneration in maize. **(A)** Diagram of *ZmDREB4.1* overexpression vector. *ZmDREB4.1* was fused to GFP and driven by CaMV 35S promoter. *Bar* gene was used as a selective marker. **(B)** The callus transformed with p35S::ZmDREB4.1:GFP. GFP fluorescence and bar protein expression were observed in this callus. However, this callus could not regenerate seedlings. **(C)** The calli with transient expression of p35S::ZmDREB4.1:GFP and p35S::GFP. The GFP fluorescence was observed in the callus which is indicated by a white pane. The arrows indicate positive calli. **(D)** The fresh weight of positive callus in **(C)**. The calli transformed with p35S::ZmDREB4.1:GFP were slightly lighter than those with p35S::GFP. The values represent the mean ± SD of the fresh weight from positive calli transformed with p35S::GFP and p35S::ZmDREB4.1:GFP vectors (for p35S::GFP, *n* = 34; for p35S::ZmDREB4.1:GFP, *n* = 32) and the statistically significant differences were evaluated by Student’s *t*-test (^∗^*P*-value < 0.05).

To further analyze the function of *ZmDREB4.1*, the full-length coding sequence of *ZmDREB4.1* fused to GFP under the control of the CaMV 35S promoter was transformed into maize immature embryo by particle bombardment (**Figure 8A**). These transformed calli were successively cultured on selection medium, recovery medium and differentiation medium. After being cultured on differentiation medium for 2 weeks, we obtained many regenerative seedlings, but they were all negative. We then examined the calli that grew slowly under a fluorescence microscope, and GFP was observed in a callus. We also detected the expression of bar protein in this callus by QUICKStix Kit (EnviroLogix, United States), and the positive band was observed. However, this callus failed to regenerate seedlings (**Figure 8B**).

In combination with the phenotypes in transgenic tobacco, we predicted that inhibition of callus regeneration might result from inhibition of callus growth and proliferation by *ZmDREB4.1* overexpression. Therefore, a callus-induction assay was performed to confirm this hypothesis. About 400 calli of similar size were transformed with p35S::ZmDREB4.1:GFP or p35S::GFP independently. The calli were then cultured on NB medium for 2 weeks. A fluorescence microscope was used to examine the GFP fluorescence. There were 34 and 32 positive calli transformed with p35S::ZmDREB4.1:GFP and p35S::ZmDREB4.1:GFP, respectively. The sizes of calli transformed with p35S::ZmDREB4.1:GFP were slightly smaller than those of the control (**Figure 8C**). The *ZmDREB4.1*-overexpressing callus was on average 52.39 mg, while this value was 69.84 mg for the control (**Figure 8D**). These results demonstrated that overexpression of *ZmDREB4.1* repressed calli growth and regeneration in maize.

## Discussion

A large number of DREB transcription factors have been reported to show a variety of roles in plant development and in response to abiotic stresses (Stockinger et al., 1997). In this study, we described the isolation and characterization of a DREB A-4 subgroup transcription factor from maize named ZmDREB4.1. *ZmDREB4.1* exhibited relatively low basal expression (**Figure 1C**), which was consistent with the expression patterns of many members of the DREB subfamily (Agarwal et al., 2017). In previous studies, several DREB A-4 subgroup genes were identified from various plant species. All these genes except for *DBF2* are induced by some abiotic stresses (Wilson et al., 1996; Kizis and Pagès, 2002; Wei et al., 2005; Huang and Liu, 2006; Bouaziz et al., 2013; Liu et al., 2015). Our results showed that the expression levels of *ZmDREB4.1* remained stable under various abiotic or biotic stresses. This expression pattern is similar to that of *DBF2*, the only DREB A-4 subgroup gene reported in maize (Kizis and Pagès, 2002). These results indicated that the regulatory mechanism of *ZmDREB4.1* expression differed from those of other reported DREB A-4 genes and may play different roles.

A dwarf phenotype was also observed in some transgenic plants, such as *DREB1B/CBF1* and *GhDREB1*-overexpressing Arabidopsis and *AtDREB1A*-overexpressing soybean (Stockinger et al., 1997; Mok and Mok, 2001; Huang et al., 2009). In the present study, we showed that *ZmDREB4.1*-overexpressing tobacco exhibited a similar phenotype, including shorter hypocotyls, stems and petioles and smaller leaves (**Figures 3A–C**). The bioactive GA contents (GA3 and GA4) in *ZmDREB4.1*-overexpressing petioles were reduced compared with those of WT (**Figure 7C**). Quantification analysis results showed that *GA2ox*, *GA3ox,* and *GA20ox* genes were all up-regulated in *ZmDREB4.1*-overexpressing tobacco (**Figure 7D**). We thus proposed that reduced endogenous GA levels were caused by increased expression levels of *GA2ox* genes, while up-regulation of *GA20ox* and *GA30ox* genes was because of the feedback mechanism in GA homeostasis regulation. The dwarf phenotype caused by overexpression of *ZmDREB4.1* could be rescued by application of exogenous GA3 (**Figure 7E**). Similar results were obtained in *DREB1B/CBF1*-overexpressing Arabidopsis and *GhDREB1*-overexpressing soybean (Stockinger et al., 1997; Huang et al., 2009). These results demonstrated that the dwarf phenotype of *ZmDREB4.1*-overexpressing tobacco might be caused by reduced contents of bioactive GAs.

In addition, our results showed that the smaller-leaf phenotype in transgenic plants could not be rescued by application of exogenous GA3. This phenomenon was also observed in *AtDREB1A*-overexpressing soybean and *GhDREB1*-overexpressing Arabidopsis (Mok and Mok, 2001; Huang et al., 2009). However, the underlying mechanisms remain unclear. The fewer number of cells and epidermal hairs (**Figure 4**) and the inhibition of callus induction (**Figure 5**) indicated that overexpression of *ZmDREB4.1* severely repressed cell division in transgenic leaves. Quantitative analysis of endogenous bioactive auxin and CK showed that the IAA, ZR, DHZR, and IPA contents were severely reduced in *ZmDREB4.1*-overexpressing tobacco (**Figure 7A**). Consistent with reduced auxin and CK levels, *YUCCA9*, *YUCCA10,* and *YUCCA11* (key enzymes in auxin biosynthesis) (Stepanova et al., 2011) as well as *IPT3* and *IPT5* (key enzymes in CK biosynthesis) (Lindner et al., 2014) were down-regulated in transgenic tobacco (**Figure 7B**). However, the application of exogenous auxin and CK could not rescue the smaller-leaf phenotype in OE9. We thus proposed that reduced auxin and CK levels might contribute to the inhibition of cell division but are not the main factor. In a word, our results demonstrated that the smaller-leaf phenotype resulted from inhibition of cell division. However, how *ZmDREB4.1* repressed cell division in leaves requires further investigation.

The DRE element bound by DREBs was involved in response to drought, low-temperature, and high-salt stresses (Yamaguchi-Shinozaki and Shinozaki, 1994). The DREB transcription factor can activate the transcription of stress-response genes by binding to the DRE element and improve plant tolerance to various stresses (Li et al., 2014). ZmDREB4.1 is a DREB transcription factor and functions as an activator (**Figures 1**, **2**). However, *ZmDREB4.1*-overexpressing tobacco exhibited reduced tolerance to dehydration stress (Supplementary Figure S6). It is reported that epidermal hairs can help plants prevent water loss (Amme et al., 2005). The reduced drought tolerance in OE9 might be due to fewer epidermal hairs. Further analysis is needed.

## Conclusion

Our results demonstrated that *ZmDREB4.1* is an important factor involved in the negative regulation of plant growth and development. We showed that ZmDREB4.1 belongs to the DREB A-4 subgroup and functions as a transcriptional activator. Overexpression of *ZmDREB4.1* repressed calli growth and regeneration in maize. *ZmDREB4.1*-overexpressing tobacco displayed a dwarf phenotype with shorter hypocotyls, stems and petioles and smaller leaves. We also showed that the smaller leaves of transgenic tobacco resulted from inhibition of cell division. The shorter hypocotyls, stems, and petioles of transgenic tobacco were caused by inhibition of cell elongation because of reduced endogenous GA levels. However, how *ZmDREB4.1* regulates leaf, hypocotyl, stem and petiole development in different means requires further investigation.

## Author Contributions

SL and JY conceived and designed the studies. SL prepared the materials, performed the experiments, analyzed the data, and wrote the original manuscript. QZ prepared the reagents. DZ participated in genetic transformation. JY revised the manuscript thoroughly and finalized the manuscript.

## Conflict of Interest Statement

The authors declare that the research was conducted in the absence of any commercial or financial relationships that could be construed as a potential conflict of interest.

## Footnotes

1 https://phytozome.jgi.doe.gov/pz/portal.html

2 https://www.ncbi.nlm.nih.gov
