# Supplementary material for: A DREB-Like Transcription Factor From Maize (Zea mays), ZmDREB4.1, Plays a Negative Role in Plant Growth and Development
Source: Front Plant Sci. 2018 Apr 4;9:395. doi: 10.3389/fpls.2018.00395 (PMC5893645; doi:10.3389/fpls.2018.00395)
Supplement: Supplementary file 1 [file Image_1.pdf]

Supplementary Materials

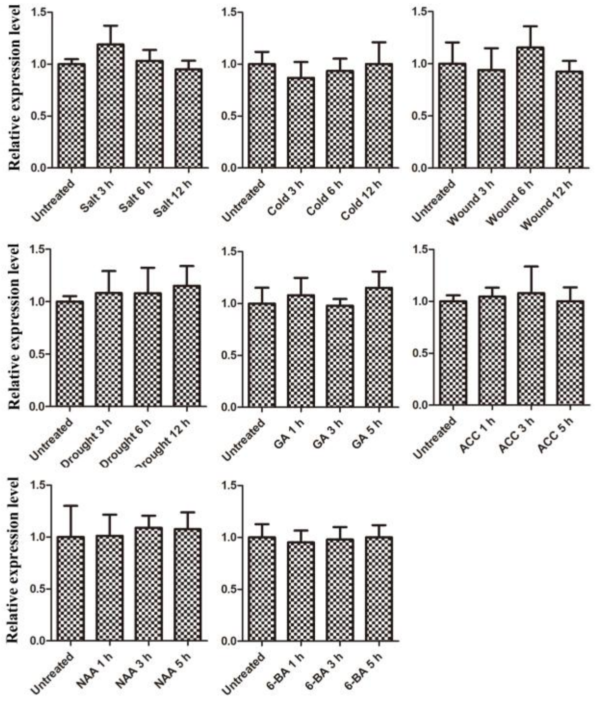

Fig. S1 The expression pattern of the *ZmDREB4.1* gene under various abiotic stresses

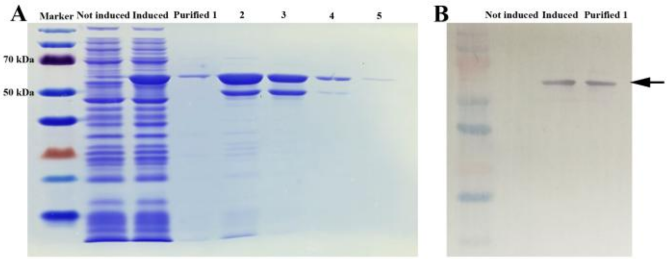

Fig. S2 MBP-ZmDREB4.1$_{31\text{-}147\ aa}$ fusion protein was obtained by the prokaryotic expression system. (A) SDS-PAGE analysis of the ZmDREB4.1$_{31\text{-}147\ aa}$ fusion protein. (B) Western blot analysis of the ZmDREB4.1$_{31\text{-}147\ aa}$ fusion protein. ZmDREB4.1$_{31\text{-}147\ aa}$ fusion protein is about 57 kDa and the black arrows indicated the target strips. The purified 1 protein was used for EMSA.

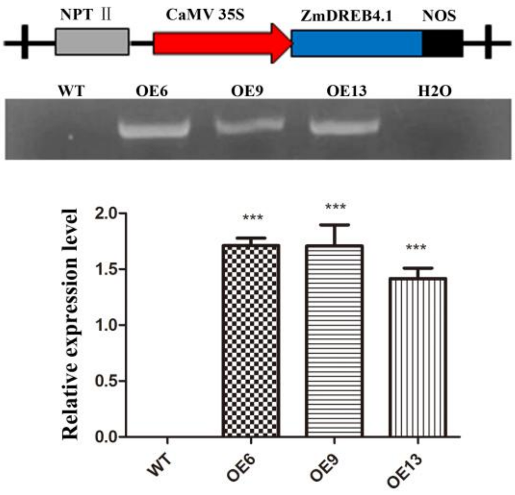

Fig. S3 Generation of *ZmDREB4.1*-overexpressing tobacco

The recombinant plasmid (p35S::ZmDREB4.1) was transformed into tobacco using the leaf disc method. PCR analysis was performed using genomic DNA as the template and primer pair ZmDREB4.1-F/R. In addition, qRT-PCR was performed using cDNA synthesized from total mRNA as template and primer pair ZmDREB4.1-qRT-F/R. The wild type was used as the control.

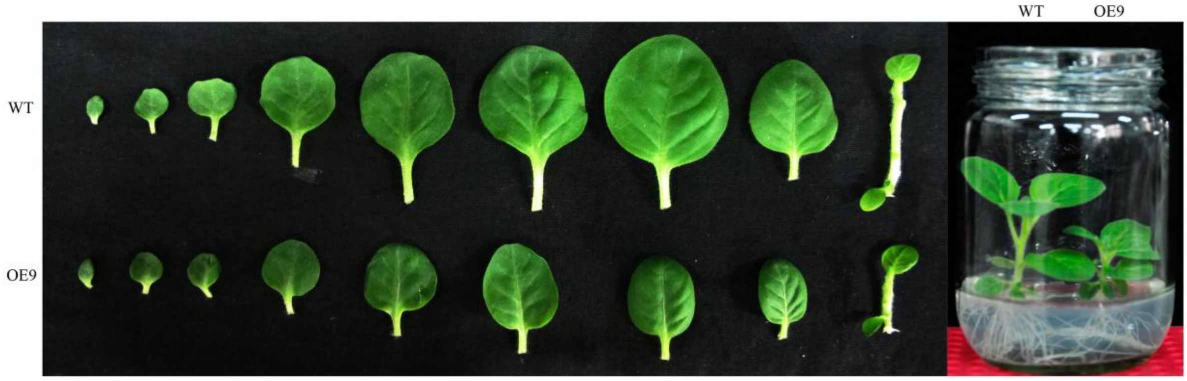

Fig. S4 Overexpression of *ZmDREB4.1* did not influence leaf number in transgenic tobacco.

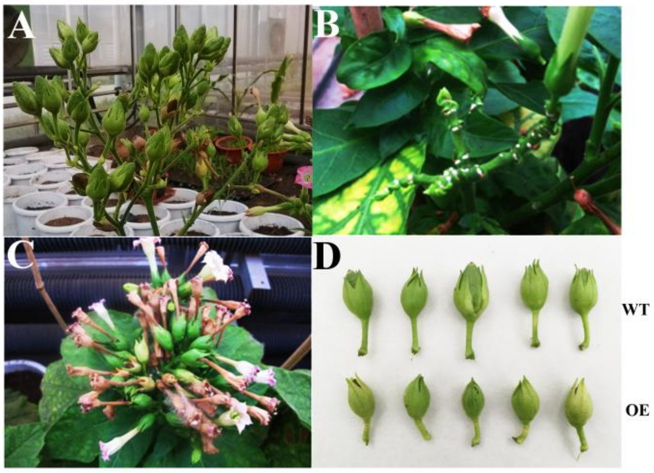

Fig. S5 Phenotype observation of *ZmDREB4.1*-overexpressing tobacco at the reproductive stage

(A) WT. *ZmDREB4.1*-overexpressing lines exhibited earlier abscission of flowers **(B)** or shorter pedicels **(C)** and **(D)**.

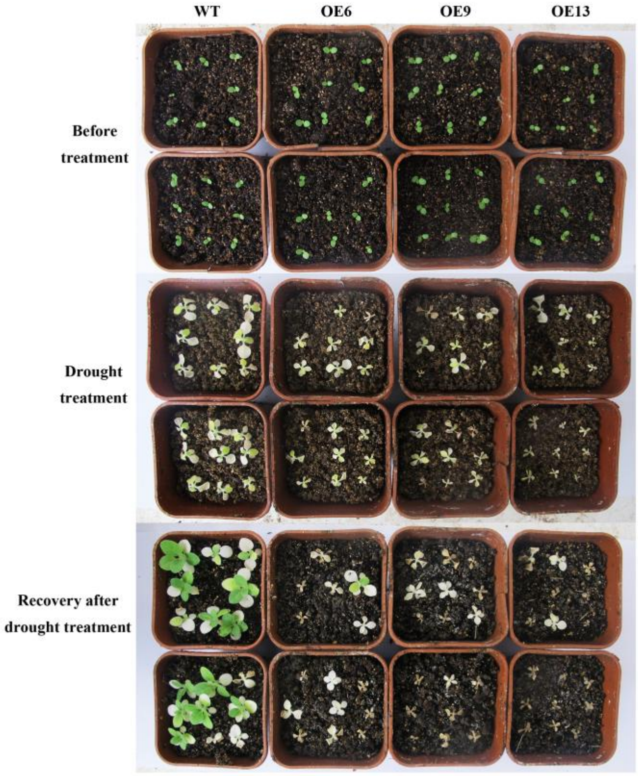

Fig. S6 Overexpression of *ZmDREB4.1* decreased drought tolerance in transgenic tobacco.
